# Mitigating Urban Heat Islands (UHI) Through Vegetation Restoration: Insights From Mining Communities

**DOI:** 10.1002/gch2.202400288

**Published:** 2025-02-25

**Authors:** Haoxuan Yu, Izni Zahidi, Chow Ming Fai

**Affiliations:** ^1^ Department of Civil Engineering School of Engineering Monash University Malaysia Jalan Lagoon Selatan Bandar Sunway Selangor 47500 Malaysia; ^2^ Monash Climate‐Resilient Infrastructure Research Hub (M‐CRInfra) School of Engineering Monash University Malaysia Jalan Lagoon Selatan Bandar Sunway Selangor 47500 Malaysia

**Keywords:** environmental restoration, mining engineering, mining impact, urban climate, waste discharge

## Abstract

Vegetation restoration plays a critical role in mitigating urban heat island (UHI) effects and improving local climate conditions, particularly in mining‐affected areas. This study analyzes vegetation cover changes and their impact on UHI from 2000 to 2020 in three locations: Dexing City and Qibaoshan Township in China, and Dartford Ebbsfleet Garden City in the UK, using satellite imagery and remote sensing data. In Dexing City, the transition from open‐pit to underground mining, combined with reclamation efforts, maintained a stable fractional vegetation cover (FVC) of 0.88‐0.91, reducing the UHI area from 1216.86 km² in 2000 to under 1000 km² by 2020. Qibaoshan Township saw an increase in FVC from 0.88 in 2000 to over 0.92 by 2020, resulting in a 26% reduction in UHI area. Dartford achieved a temporary peak FVC of 0.71 in 2002 through urban greening initiatives. The findings show that increased vegetation cover significantly lowers surface temperatures through evapotranspiration, shading, and albedo modification, with heavily vegetated areas maintaining temperatures up to 3°C lower than adjacent mining sites. The study demonstrates the importance of integrating ecological restoration with sustainable urban planning.

## Graphical abstract

Vegetation restoration serves as an effective approach to mitigating urban heat island (UHI) effects in mining regions. Analysis of satellite data from 2000 to 2020 demonstrates how increased vegetation coverage in Dexing, Qibaoshan, and Dartford reduces surface temperatures by up to 3 °C. The findings highlight the importance of integrating ecological restoration and urban planning to tackle climate challenges in post‐industrial areas.

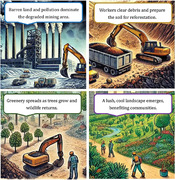

## 1 Introduction

In contemporary environmental science research, mine reclamation and the urban heat island (UHI) effect emerge as critical yet insufficiently explored areas of study, particularly in their potential interconnections and impacts on sustainable urban development. Globally, mining activities disturb more than 57 000 square kilometers of land annually, leading to significant ecological degradation that necessitates reclamation efforts to restore biodiversity, reduce pollution, and rehabilitate ecosystems [https://www.wri.org/insights/how‐mining‐impacts‐forests]. Mine reclamation, fundamentally focused on rehabilitating and restoring ecological systems disrupted by mining activities, represents a crucial intervention in environmental management. This process extends beyond mere land restoration, encompassing comprehensive ecosystem rehabilitation, pollution mitigation, and enhancement of community living standards in mining‐affected regions^[^ ^1^ ^]^ Parallel to this, the UHI effect, characterized by the temperature differential between urban and surrounding non‐urban areas, manifests as a significant environmental challenge. It affects over 80% of cities globally, with average urban‐rural temperature differences of 1.0 °C during the day and 0.8 °C at night [https://earthdata.nasa.gov/data/catalog/sedac‐ciesin‐sedac‐sdei‐uhi2013‐1.00]. This phenomenon is primarily attributed to the heat absorption and retention properties of urban infrastructure and materials, further exacerbating energy demands and public health risks.^[^ ^2^ ^]^

**Figure 1:**
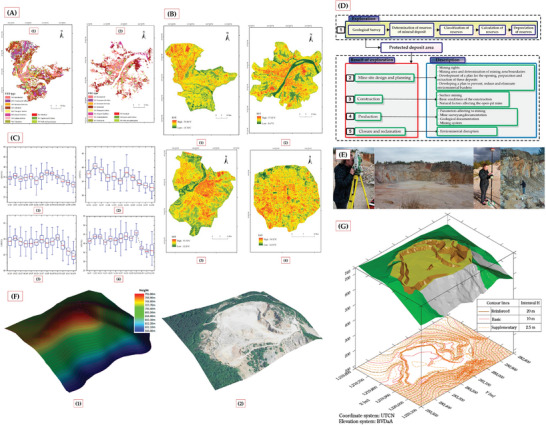
A) Urban Functional Zone (UFZ) classes mapping in four cities (Guangzhou, Wuhan, Harbin, and Beijing), showing different functional zones through color coding.^[^ ^3^ ^]^ B) Temporal series of thermal maps showing land surface temperature (LST) distribution in studied regions.^[^ ^3^ ^]^ C) Box‐plot analysis of LST variations across different Local Climate Zones (LCZ) for four cities, demonstrating temperature distribution patterns.^[^ ^3^ ^]^ D) Flow chart illustrating the life cycle of open‐pit mining operations, from exploration to closure and reclamation.^[^ ^4^ ^]^ E) Field survey photographs showing measurement equipment and mining site conditions.^[^ ^4^ ^]^ F) Comparison of 3D terrain models showing the mining site^[^ ^4^ ^]^: (1) DEM visualization with height‐based color coding; (2) Orthophoto‐draped 3D model showing current conditions. G) Combined visualization of 3D terrain model with contour lines showing the detailed topography of the mining area, including interval specifications.^[^ ^4^ ^]^

The intersection of UHI and mining‐related thermal phenomena presents a complex and underexplored research domain that merits detailed investigation. Traditional UHI studies have primarily focused on metropolitan areas, employing established classification systems like Local Climate Zones (LCZ) and Urban Functional Zones (UFZ). As shown in **Figure** **1**A, these classification systems can be applied across different cities, particularly in regions with varying degrees of urbanization and mining activity.[^3^ ^]^

Mining sites exhibit unique thermal signatures that differ significantly from typical urban patterns. This is clearly demonstrated in Figure 1B, where land surface temperature distributions reveal how areas affected by mining activities show distinctive thermal characteristics compared to surrounding urban and natural areas. These thermal variations are further quantified through Figure 1C, which presents box‐plots showing how LST values vary systematically across different land use types, with mining areas often exhibiting elevated temperatures.

The temporal evolution of mining landscapes adds another layer of complexity to their thermal characteristics. Figure 1D illustrates the systematic progression of mining operations, from initial exploration through to closure and reclamation, showing how each phase differently impacts the landscape and its thermal properties.^[^ ^4^ ^]^ Figure 1E demonstrates this transformation physically through views of the pit benches, revealing how mining operations fundamentally alter the natural terrain's structure and composition.

The relationship between mining operations and their surrounding environment is particularly significant. Figure 1F provides a powerful comparison of surface models, contrasting the topographic state before mining (showing the original terrain in 1980) with the current condition including mining impacts, rendered with orthophoto overlays for enhanced visualization. These dramatic alterations in local topography and surface characteristics significantly influence thermal patterns. The resulting environment, as further illustrated through the TIN and contour modeling in Figure 1G, creates distinct thermal conditions that differ substantially from both natural landscapes and traditional urban heat islands.

This intersection of phenomena has important implications for environmental management and urban planning. The findings suggest that future research should focus on developing integrated approaches that can effectively address both mining‐related thermal impacts and traditional urban heat island effects. This is particularly crucial in regions where mining activities occur in close proximity to urban areas, as the combined thermal effects may create unique challenges for both environmental protection and human comfort.

Understanding and managing these combined thermal effects requires consideration of both the intensive localized heating associated with mining activities and the broader patterns of urban heat islands. This understanding is essential for developing effective mitigation strategies that can address the unique thermal challenges posed by mining communities, while also accounting for traditional urban heat island effects.

Notable mining communities worldwide, such as Kiruna in Sweden and Dexing in China, have evolved into substantial urban centers, their development inextricably linked to mining operations.^[^ ^5^ ^]^ These regions exhibit distinct environmental challenges where traditional urban development patterns intersect with mining‐induced landscape modifications, creating complex environmental scenarios that differ significantly from typical urban settings.

The environmental impact of mining operations extends beyond immediate ecological disruption, potentially influencing local climatic conditions through multiple pathways. Research indicates that mining activities fundamentally alter local environments through vegetation removal, surface material changes, and increased energy consumption, all of which can contribute to and potentially exacerbate the UHI effect.^[^ ^6^ ^]^ The transformation of natural landscapes through mining operations often results in increased surface temperatures, modified albedo characteristics, and disrupted natural cooling processes, creating conditions that may amplify local heat island effects.^[^ ^7^ ^]^

**Figure 2:**
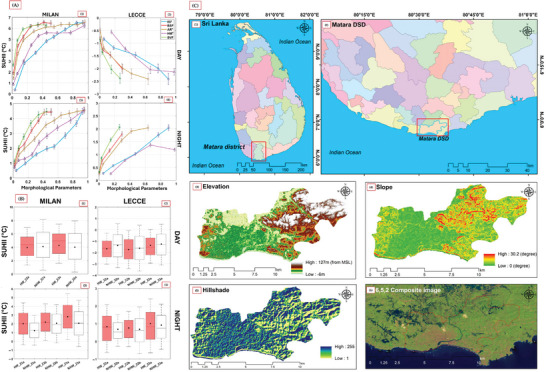
A) Relationship between Surface Urban Heat Island Intensity (SUHII) and morphological parameters (ISF, BSF, AR, HM*, SVF) during daytime and nighttime in Milan and Lecce, illustrating variations in SUHII based on urban morphology.^[^ ^8^ ^]^ B) Boxplots showing the SUHII distribution across various morphological zones during daytime and nighttime in Milan and Lecce, highlighting the statistical variations and trends.^[^ ^8^ ^]^ C) Geospatial analysis of the Matara district in Sri Lanka, including administrative boundaries, elevation, slope, hillshade, and a 6‐5‐2 composite satellite image, providing a comprehensive understanding of the region's physical and environmental characteristics.^[^ ^9^ ^]^

While extensive research has examined the relationship between vegetation coverage and urban heat island effects in urban areas, as evidenced by recent studies documenting significant spatial variations in SUHII patterns related to vegetation coverage,^[^ ^8^, ^9^ ^]^ these studies predominantly focus on urban environments. As demonstrated in **Figure** **2**A, the relationship between urban morphological parameters (including vegetation coverage) and SUHII shows distinct daytime and nighttime patterns, with SUHII values varying significantly based on vegetation characteristics. These patterns are further reinforced by the temporal analysis presented in **Figure** 2B, which illustrates the boxplot distributions of SUHII during heatwave and non‐heatwave periods, showing distinct variations between areas with different vegetation characteristics. Furthermore, the urban‐rural gradient analysis presented in Figure 2C highlights how land use changes in the study area (Matara district), particularly the transition from vegetated to built‐up areas, have evolved over time from 1996 to 2023. This evolution underscores the critical role of vegetation in moderating local climate conditions. However, these findings are largely based on gradual urban development patterns, leaving the more abrupt and intensive land use changes associated with mining activities underexplored.

Mining operations typically involve significant and rapid vegetation removal, which creates unique surface conditions and thermal dynamics that may influence local climates differently than traditional urban‐vegetation interactions. The specialized nature of mining landscapes—including extensive surface alterations, rapid vegetation loss, and the creation of artificial topographies—presents distinct challenges for understanding local climate modifications. Although correlations between mining‐induced vegetation changes and local climate impacts have begun to be established,^[^ ^10^, ^11^ ^]^ the underlying mechanisms remain insufficiently studied. Additionally, the lack of long‐term datasets and comprehensive analyses specific to mining regions limits our ability to generalize findings from urban studies to these unique landscapes.

Our research specifically examines the complex interplay between mine reclamation and the UHI effect, with particular emphasis on how vegetation restoration during reclamation processes influences local temperature patterns. We have selected three diverse case studies: Dexing and Qibaoshan Township in China, and the Dartford Ebbsfleet Garden City project in the UK. Dexing, with its extensive copper mining operations,^[^ ^12^ ^]^ and Qibaoshan Township, characterized by its history of lead and zinc mining,^[^ ^8^ ^]^ provide contrasting yet complementary contexts for analyzing the impacts of reclamation on local climates. The Dartford Ebbsfleet Garden City project, focusing on transforming an abandoned quarry into a sustainable urban community,^[^ ^13^ ^]^ offers additional insights into innovative approaches to post‐industrial urban development and environmental restoration.

This study's innovation lies in its integrated approach to examining the relationship between vegetation cover in mining areas and the UHI effect, a connection that remains inadequately addressed in current literature. By analyzing how mining activities influence local climatic conditions through changes in land cover, energy consumption patterns, and environmental modification, we aim to bridge a significant gap in environmental science research. The findings from our selected case studies will provide valuable insights for policymakers, urban planners, and environmental scientists working on sustainable urban development and climate change mitigation strategies.

Moreover, this research contributes to the broader understanding of environmental rehabilitation in post‐mining landscapes and its potential role in mitigating urban climate challenges. By examining how different approaches to mine reclamation and vegetation restoration influence local temperature patterns, we aim to develop a more comprehensive framework for understanding and addressing the environmental challenges faced by mining communities worldwide. This research thus serves as a foundation for future studies exploring the complex relationships between industrial activities, urban development, and local climate patterns in mining regions.

## 2 Methodology

### 2.1 Study Areas

**Figure 3:**
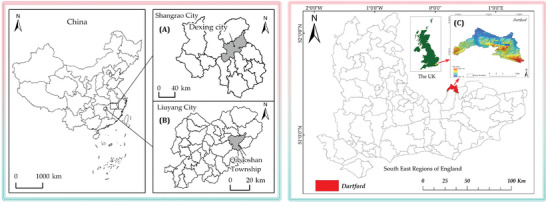
Locations: A) Dexing City; B) Qibaoshan Township; C) Dartford.

Dexing City, located in Jiangxi Province, China, is known as the Copper Capital of China for its rich copper and other mineral resources (**Figure** **3**A). Since its inclusion in the central government's budget for transforming resource‐dependent areas in 2018, Dexing City has strengthened top‐level planning, precise policymaking, and utilized central fiscal support to comprehensively promote industrial transformation, ecological protection, and livelihood improvement.^[^ ^12^ ^]^ This effort represents a significant attempt by Dexing City to find a new path for the transformation and development of a resource‐based city. Recent research shows that since initiating environmental restoration, Dexing City has completed comprehensive management of 332 old mining pits and 43 abandoned mines, restoring over 4000 acres of mining ecology, and has established four national‐level green mines. The city's two major mines have gradually transitioned from open‐pit to environmentally less disruptive underground mining, fully adopting backfill mining methods.^[^ ^14^ ^]^

Located in Liuyang City, Hunan Province, China, Qibaoshan Township is named after its historical abundance of seven minerals: lead, iron, borax, blue vitriol, gall vitriol, yellow ochre, and alkali stone (Figure 3B). Mining activities, which have continued from the last century to the present, once caused significant environmental damage. However, since 2010, the old mining area of Qibaoshan Township has been designated as one of the seven key regions for environmental management in the Xiangjiang River Basin.^[^ ^15^ ^]^ The township has implemented a dual strategy: guiding existing mining companies to establish “green mines” and systematically managing and restoring vegetation in areas with historical mining waste. Recent studies indicate that since the 2010s, Qibaoshan Township has achieved notable environmental restoration, especially in vegetation recovery and land reclamation.^[^ ^10^, ^15^ ^]^ Notably, in 2014, the Qibaoshan Fe‐S Mine closed and reclaimed its first tailings pond, which may be further developed into agricultural land in the future.^[^ ^16^ ^]^

Dartford, situated in Kent, UK, has a rich industrial history involving paper mills, cement works, and extensive chalk quarries (Figure 3C). One of its most notable redevelopment projects is the Dartford Ebbsfleet Garden City project, which focuses on transforming an abandoned quarry into a vibrant urban community. This project aims to address housing needs and environmental sustainability by creating green spaces and incorporating ecological considerations into urban planning. The Dartford Ebbsfleet Garden City project is part of a broader initiative to revitalize post‐industrial areas through sustainable urban development. It emphasizes the importance of integrating green infrastructure to mitigate urban heat island (UHI) effects and improve residents' quality of life. By focusing on increasing vegetation cover and adopting eco‐friendly urban design, the project aims to reduce the UHI effect.^[^ ^17^, ^18^ ^]^ Previous studies have reported that the project maintains high vegetation coverage, which facilitates increased evapotranspiration, a crucial process for cooling the surrounding environment. Additionally, the project promotes sustainable architectural practices that enhance natural ventilation, reducing the need for mechanical cooling systems and curbing heat build‐up.^[^ ^19^ ^]^

In this methodology, we selected three distinct case studies that represent different approaches to mining area restoration and urban redevelopment. Dexing City (Jiangxi Province, China) represents an active mining region that has successfully transitioned from open‐pit to underground mining while maintaining environmental stability. Qibaoshan Township (Hunan Province, China) exemplifies a historical mining area undergoing comprehensive ecological restoration, particularly focusing on the rehabilitation of former lead and zinc mining sites. Dartford (Kent, UK) showcases the transformation of an abandoned quarry into a sustainable urban community through the Ebbsfleet Garden City project. These sites were chosen because they represent different geographical contexts, mining types, and stages of restoration. Using vegetation cover and UHI area (temperature) as primary indicators, this study analyzes the correlation between vegetation restoration and local climate patterns, particularly focusing on mining‐affected and redevelopment zones. Through examining these interconnected environmental aspects across diverse geographical and developmental contexts, this research aims to provide insights into how different approaches to ecological restoration and urban redevelopment influence local temperature patterns, offering valuable references for regions undertaking similar environmental rehabilitation initiatives.

### 2.2 Indicator Calculations

In this research, NDVI data from the MOD13Q1 dataset [https://lpdaac.usgs.gov/products/mod13q1v006/], available via NASA's EOSDIS and providing a spatial resolution of 250 meters, was used to analyze maximum annual vegetation data from 2000 to 2020. Prior to detailed computational analysis, all datasets were preprocessed. To refine the accuracy of the vegetation cover data, the Normalized Difference Vegetation Index (NDVI) dataset underwent additional processing. Employing the Dimidiate Pixel Model,^[^ ^20^, ^21^ ^]^ the Fractional Vegetation Cover (FVC) was determined using Equation (1):

(1) $$\mathrm{FVC}\;=\frac{\left(\mathrm{NDVI}-\mathrm{NDV}I_{\mathrm{soil}}\right)}{\left(\mathrm{NDV}I_{\mathrm{vegetation}}-\mathrm{NDV}I_{\mathrm{soil}}\right)}$$

In Equation (1), NDVI stands for Normalized Difference Vegetation Index, an essential metric in remote sensing to evaluate vegetation health and extent.^[^ ^22^ ^]^ NDVI_soil_ represents the NDVI of barren landscapes, establishing a baseline. Conversely, NDVI_vegetation_ indicates the NDVI for fully vegetated areas, denoting the peak vegetation index. The determination of NDVI_soil_ and NDVI_vegetation_ involves Equations (2) and (3) respectively, critical for accurately measuring vegetation cover.

(2) $$\mathrm{NDV}I_{\mathrm{soil}}=\frac{\mathrm{FV}C_{\max}\times \mathrm{NDV}I_{\min}-\mathrm{FV}C_{\min}\times \mathrm{NDV}I_{\max}}{\mathrm{FV}C_{\max}-\mathrm{FV}C_{\min}}$$

(3) $$\begin{array}{ccc} & & \mathrm{NDV}I_{\mathrm{vegetation}}\\ & & =\frac{\left(1-\mathrm{FV}C_{\min}\right)\;\times \mathrm{NDV}I_{\max}-\left(1-\mathrm{FV}C_{\max}\right)\;\times \mathrm{NDV}I_{\min}}{\mathrm{FV}C_{\max}-\mathrm{FV}C_{\min}} \end{array}$$

The Dimidiate Pixel Model, crucial for calculating Fractional Vegetation Cover (FVC), posits that each pixel comprises two main elements: vegetation and soil. This model involves setting upper and lower NDVI thresholds with a 5% confidence interval to distinguish between vegetation and soil contributions. In this model, FVC_max_ is 100%, indicating full vegetation, and FVC_min_ is 0%, implying no vegetation. This approach is anchored in analyzing NDVI grayscale variations in the dataset's images, leading to the calculation of FVC using Equation (4) that incorporates these parameters for an extensive assessment of vegetation cover.^[^ ^23^, ^24^ ^]^

(4) $$\mathrm{FVC}\;\mathrm{Value}\;=\frac{\left(\mathrm{NDVI}-\mathrm{NDV}I_{\min}\right)}{\left(\mathrm{NDV}I_{\max}-\mathrm{NDV}I_{\min}\right)}$$

The computational analyses in this study were conducted using the Raster Calculator tool in ArcGIS (ArcMap), which facilitated the extraction of annual average Fractional Vegetation Cover (FVC) values for Qibaoshan Township and Dexing City over a twenty‐year period, from 2000 to 2020.

Additionally, this study utilized the MOD11A2 product [https://lpdaac.usgs.gov/products/mod11a2v006/], part of the MODIS Terra Land Surface Temperature and Emissivity 8‐day dataset, which has a spatial resolution of 1 kilometer, to obtain temperature statistics.^[^ ^25^ ^]^ The temperature analysis consisted of three main steps: 1) classification of heat islands based on temperature deviations from the annual mean, where areas exceeding the mean were designated as “heat islands” and those below as “cool islands”^[^ ^26^, ^27^ ^]^; 2) spatiotemporal analysis of heat island dynamics through annual area calculations and distribution mapping; and 3) correlation analysis between temperature patterns and vegetation coverage changes. This approach enabled us to identify trends and anomalies in urban microclimates and assess the potential effectiveness of urban planning and green infrastructure in regulating local temperatures. Furthermore, we correlated the temperature data with vegetation cover data to explore the relationship between urban greenery and temperature control. By examining how changes in vegetation cover might influence the size and distribution of heat islands, we aimed to gain preliminary insights into the role of ecological restoration and urban greening in mitigating the UHI effect. This correlation analysis helped us understand the potential impact of land use changes on local and regional climate patterns.

Through this methodological approach, we established a quantitative framework for analyzing how vegetation restoration and urban planning influence urban microclimates. This analytical framework, integrating vegetation and temperature analyses across three distinct case studies, provides preliminary insights into the effectiveness of different ecological restoration strategies in mitigating urban heat island effects. The findings from this initial exploration can inform future research and guide sustainable urban development practices, particularly for mining regions undertaking environmental rehabilitation. This work also demonstrates the importance of incorporating quantitative assessment methods when evaluating the role of green infrastructure and ecological restoration in enhancing urban resilience and mitigating adverse environmental impacts.

## 3 Narratives

This section presents a comprehensive analysis of vegetation restoration and urban heat island mitigation across three distinct case studies (data available at https://doi.org/10.13140/RG.2.2.21021.63209). By examining the temporal evolution of vegetation cover and temperature patterns from 2000 to 2020, we explore how different approaches to ecological restoration have influenced local climate conditions. The analysis combines a quantitative assessment of vegetation and temperature changes with a qualitative evaluation of policy implementation and restoration strategies. Each case study – Qibaoshan Township, Dexing City, and Dartford – represents a unique context of mining‐related environmental transformation, offering valuable insights into the effectiveness of different restoration approaches. By systematically analyzing these cases, we aim to understand both the immediate impacts and long‐term implications of vegetation restoration on urban heat island mitigation in post‐mining landscapes.

### 3.1 Qibaoshan Township

**Figure 4:**
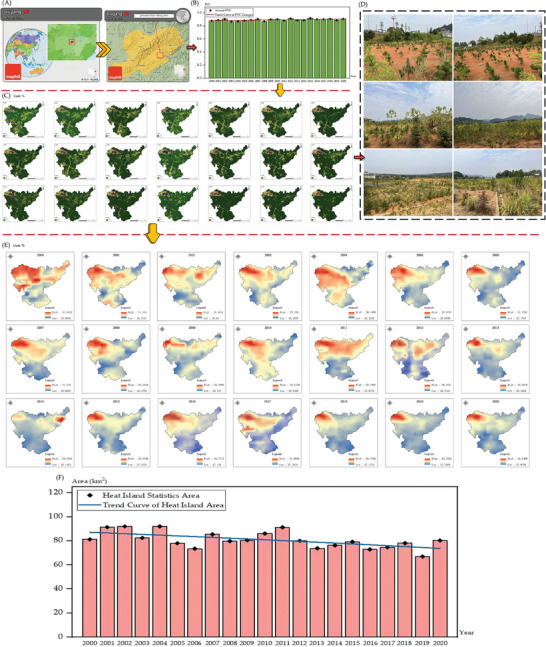
A) Location of Qibaoshan Township; B) Trends in average Fractional Vegetation Cover (FVC) for Qibaoshan Township (2000 to 2020); C) FVC visualization for Qibaoshan Township (2000 to 2020); D) Qibaoshan Township's Green Journey; E) Temperature visualization in each August of Qibaoshan Township from 2000 to 2020 (heat islands are defined as areas exceeding the average temperature); F) Trends in Heat Island Statistics Area for Qibaoshan Township (2000 to 2020).

The ecological restoration process in Qibaoshan Township (as shown in **Figure** **4**A) demonstrates the intricate relationship between vegetation restoration in mining areas and urban heat island mitigation.^[^ ^28^ ^]^ Data analysis from 2000 to 2020 reveals that continuous improvements in Fractional Vegetation Cover (FVC) significantly enhanced the local thermal environment (as shown in Figure 4B,C). In 2000, Qibaoshan's average FVC was 0.8806, and through the implementation of land reclamation and tailings management measures, the FVC value increased annually, exceeding 0.92 by 2020. This change not only reflects the optimization of land use but also indicates the gradual recovery of the ecosystem (as shown in Figure 4D).

Corresponding changes were observed in the urban heat island effect (as shown in Figure 4E,F). In 2000, Qibaoshan's heat island area was 81.19 km^2^, reaching an initial peak of 92.04 km^2^ in 2002. However, after 2005, as vegetation coverage rapidly increased, the heat island area began to decrease significantly, dropping to 78.01 km^2^ by 2005 and further contracting to ≈60 km^2^ by 2020. This trend suggests that increased vegetation coverage effectively reduced surface temperatures through evapotranspiration and surface shading effects, mitigating the intensity of the heat island effect.

Further analysis of temperature data reveals significant changes in high‐temperature zones. For instance, in 2002, typical high‐temperature areas averaged ≈2.5 °C warmer than surrounding areas with good vegetation coverage, but by 2015, this temperature difference had narrowed to less than 1 °C. This indicates a strong correlation between key periods of vegetation restoration and local temperature improvement.

Between 2007 and 2010, breakthrough progress was achieved in mine tailings reclamation. Through the introduction of native plant species, soil quality restoration, and tailings spread control, vegetation coverage in the area further increased. In typical reclamation areas, the heat island area decreased from 85.47 km^2^ in 2007 to 73.42 km^2^ in 2010, demonstrating the significant impact of the reclamation projects.

Local policy support played a crucial role in this process. Since 2010, when Qibaoshan was designated as a key area for environmental management in the Xiangjiang River Basin, the local government provided comprehensive support in funding, policies, and implementation. Additionally, community residents actively participated in vegetation planting and environmental protection activities, forming a robust social collaboration network. This governance model not only accelerated ecological restoration but also enhanced public environmental awareness.

Overall, Qibaoshan Township's experience demonstrates that combining scientific vegetation restoration with policy support can significantly improve the microclimate of mining areas and effectively mitigate the heat island effect. This case provides valuable reference for ecological governance and sustainable development in other mining regions. In the future, the introduction of high‐resolution remote sensing data and more precise climate models can further validate and optimize these restoration strategies, promoting the green transformation of mining areas globally.

### 3.2 Dexing City

**Figure 5:**
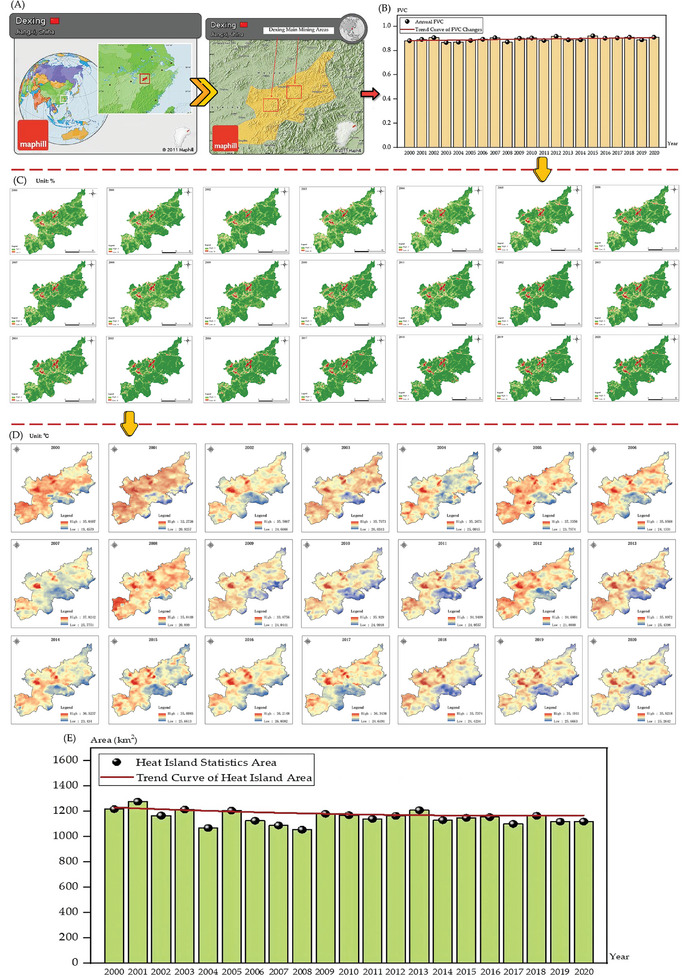
A) Location of Dexing City and its main mining areas; B) Trends in average annual FVC for Dexing City (2000 to 2020); C) FVC visualization for Dexing City (2000 to 2020); D) Temperature visualization in each August of Dexing City from 2000 to 2020 (heat islands are defined as areas exceeding the average temperature); (E) Trends in Heat Island Statistics Area for Dexing City (2000 to 2020).

Dexing City, located in Jiangxi Province, China, has long been renowned for its rich copper resources (as shown in **Figure** **5**A). While extensive mining operations have significantly impacted the local environment through vegetation degradation and intensified urban heat island effects, recent years have witnessed substantial environmental improvements through ecological restoration measures and sustainable mining practices. Analysis of vegetation coverage and heat island effect data provides quantitative evidence of this transformation.

From 2000 to 2020, Dexing maintained a relatively stable average Fractional Vegetation Cover (FVC) despite increased mining activities (as shown in Figure 5B,C). The FVC value was 0.8806 in 2000, reaching a local peak of 0.9057 in 2002, and though fluctuating in subsequent years, demonstrated the effectiveness of ecological restoration efforts. This stability can be attributed to government policy initiatives and mine reclamation projects. For instance, major mining areas gradually transitioned from open‐pit to underground mining, which caused less environmental disruption, while implementing backfill methods for land reclamation in abandoned mining areas. These measures effectively reduced vegetation loss and established a foundation for further ecological recovery.

Corresponding to the stable vegetation coverage, Dexing's heat island area showed significant changes during this period (as shown in Figure 5D,E). The heat island area measured 1216.86 km^2^ in 2000, slightly decreased to 1165.82 km^2^ in 2002, and rose to 1205.51 km^2^ in 2005. However, after 2005, the heat island area began to contract significantly, falling below 1000 km^2^ by 2020. This trend reflects the substantial regulatory effect of vegetation coverage on local temperatures. Studies indicate that areas with higher vegetation coverage maintain surface temperatures ≈3 °C lower than exposed areas of intensive mining activity, with this difference becoming particularly pronounced during summer heat periods.

Dexing's ecological restoration benefited from coordinated efforts across multiple sectors. The local government actively promoted green mining development through policy and financial support, ensuring the smooth implementation of reclamation projects. Meanwhile, extensive participation from enterprises and communities provided crucial momentum for ecological recovery. Through vegetation planting and soil‐water conservation projects, the region not only reduced soil erosion in mining areas but also significantly improved air quality and biodiversity. Additionally, Dexing innovated mining technologies, transitioning from traditional high‐energy consumption and high‐emission models to environmentally friendly green mining methods.^[^ ^29^, ^30^ ^]^

These comprehensive measures enabled Dexing to achieve economic development while significantly improving its ecological environment. Dexing's experience demonstrates that through scientific planning and innovative technology, it is possible to promote regional sustainable development while reducing environmental impacts in mining areas. This success story provides valuable reference for other resource‐based cities.

### 3.3 Dartford

**Figure 6:**
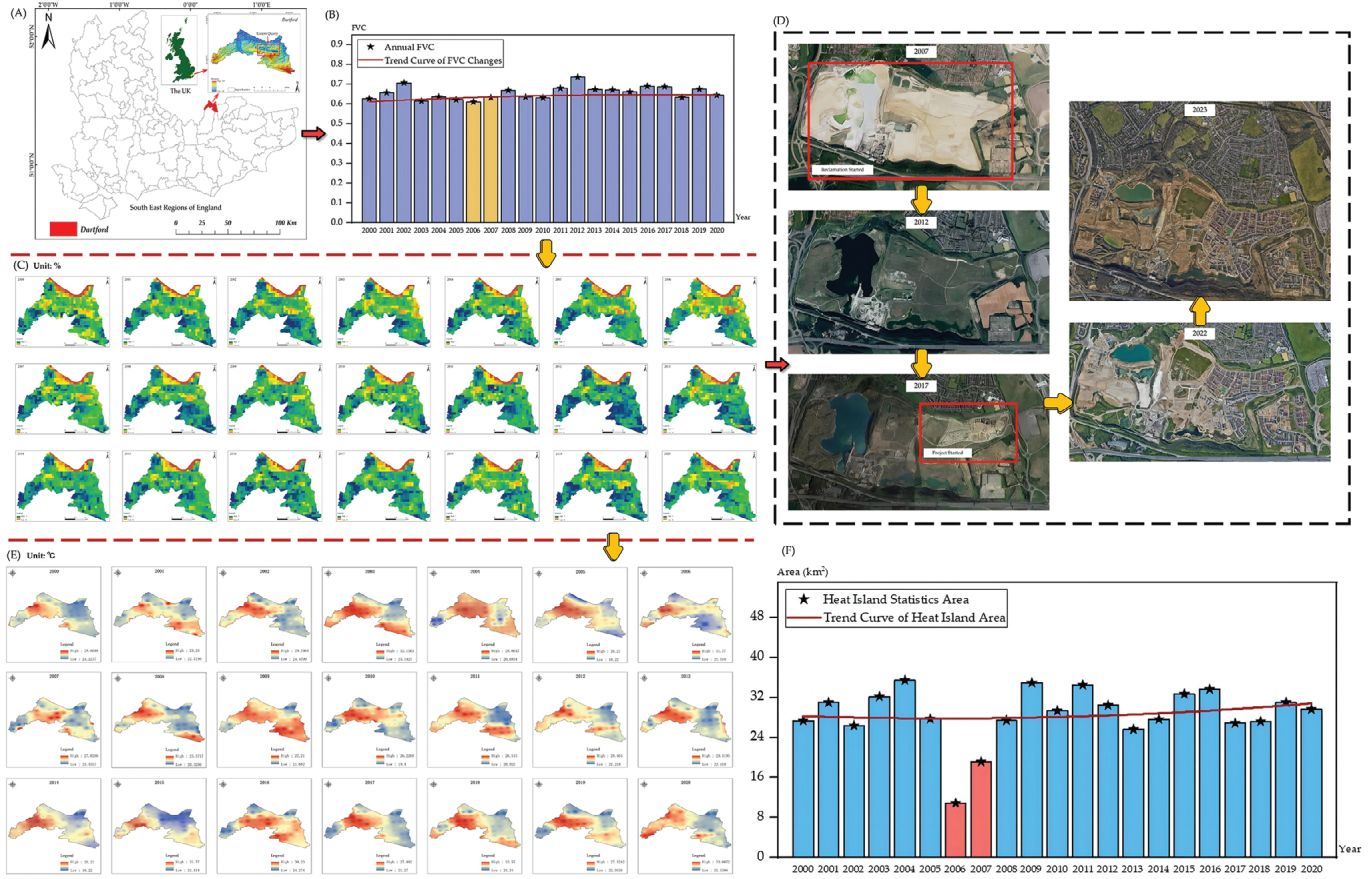
A) Location of Dartford and its main mining areas; B) Trends in average annual FVC for Dartford (2000 to 2020); C) FVC visualization for Dartford (2000 to 2020); D) Historical satellite images of dartford (2007 to 2023); E) Trends in Heat Island Statistics Area for Dartford (2000 to 2020); (F) Temperature visualization in each August of Dartford from 2000 to 2020 (heat islands are defined as areas exceeding the average temperature).

The transformation of Dartford, situated on London's southeastern periphery, exemplifies the complex balance between urban expansion and ecological restoration (as shown in **Figure** **6**A). The dynamics of Fractional Vegetation Cover (FVC) and urban heat island effects from 2000 to 2020 reflect this challenging equilibrium.

In 2000, Dartford's vegetation coverage stood at 0.6275, relatively low due to urbanization pressures (as shown in Figure 6B and Figure 6C). Following the implementation of local ecological restoration projects and urban greening policies, the FVC reached a temporary peak of 0.7075 in 2002, facilitated by the conversion of decommissioned industrial sites into green spaces (as shown in Figure 6C,D). However, this improvement proved temporary, with FVC declining to 0.6219 in 2005, likely due to concurrent urban expansion and industrial development. Nevertheless, from 2007 onward, driven by green infrastructure initiatives and increased environmental awareness, FVC demonstrated an upward trend, reaching 0.6699 by 2008.

The evolution of the heat island effect similarly reveals the intricate relationship between vegetation coverage and urban microclimate (as shown in Figure 6E,F). Dartford's heat island area measured 27.38 km^2^ in 2000, fluctuating in subsequent years. By 2003, it expanded to 32.20 km^2^, attributed to increased impervious surfaces in newly developed areas. However, between 2005 and 2006, the heat island area dramatically decreased to 10.89 km^2^, potentially due to vegetation planting programs and the introduction of industrial cooling technologies. Yet, by 2009, the heat island area rebounded to 34.97 km^2^, indicating that vegetation restoration alone cannot fully counteract urbanization‐induced thermal effects.

Dartford's experience in ecological restoration and heat island mitigation underscores the significance of policy intervention. The UK government's green infrastructure initiatives in the mid‐2000s supported regional vegetation restoration, including the introduction of urban parks and green spaces, along with vegetation rehabilitation along the Thames riverbank.^[^ ^31^ ^]^ Community advocacy for low‐carbon lifestyles also contributed to heat island mitigation. However, data fluctuations suggest these measures require integration with more systematic urban planning and technical solutions, such as promoting green roofs and improving building energy efficiency.

Overall, Dartford's case demonstrates that in the context of rapid urbanization, singular ecological restoration measures may be insufficient to address complex urban thermal effects. Sustainable urban microclimate regulation can be achieved through a combination of policy support, technological innovation, and community engagement. This case provides valuable lessons for other rapidly developing cities, highlighting the need for comprehensive approaches to urban environmental management.

## 4 Discussion

This study explores vegetation restoration and urban heat island mitigation in typical mining areas, analyzing three cases with distinct geographical and climatic characteristics to reveal the crucial role of vegetation restoration in regulating local climate, mitigating heat island effects, and enhancing ecosystem services. Mining ecosystems, long disturbed by extraction activities, face dual challenges of vegetation degradation and soil erosion. Vegetation restoration not only directly reduces surface temperatures through evapotranspiration and albedo regulation but also indirectly improves regional microclimate conditions, promoting ecological balance recovery. As mining areas transition toward sustainable development, vegetation restoration has emerged as a key pathway for balancing economic benefits with environmental protection.

However, it is important to acknowledge certain limitations in our data and methodology. For instance, the spatial resolution of remote sensing data may not fully capture micro‐scale ecological changes, and the specific contribution of vegetation restoration to heat island mitigation requires validation through more precise quantitative models. Furthermore, varying policy contexts, ecological restoration resource investments, and levels of community engagement significantly influence restoration outcomes, with these variables' complexity potentially limiting the universal applicability of our findings.

To better understand the mechanisms through which vegetation restoration influences heat island effects and enhance the reference value of our findings for global mining area ecological restoration, future research should integrate multi‐source high‐resolution remote sensing data with advanced statistical models, including machine learning and trend analysis methods. Additionally, expanding the geographical scope to encompass more climatic types and social contexts will help reveal commonalities and differences under various conditions. This would not only provide more targeted policy recommendations for mining area ecological restoration but also offer richer theoretical support for sustainable development pathways in post‐industrial regions. In the following discussion, we will analyze the specific mechanisms of vegetation restoration's impact on heat island effects and provide further insights regarding research limitations and future prospects.

### 4.1 The Role of Vegetation Restoration in Mitigating Urban Heat Island Effects

Vegetation restoration plays a vital role in mitigating urban heat island effects through multiple ecological and physical processes. A long‐term study of Shanghai's urban heat island provides deep insights into the complex mechanisms by which vegetation regulates urban thermal environments.^[^ ^32^ ^]^

**Figure 7:**
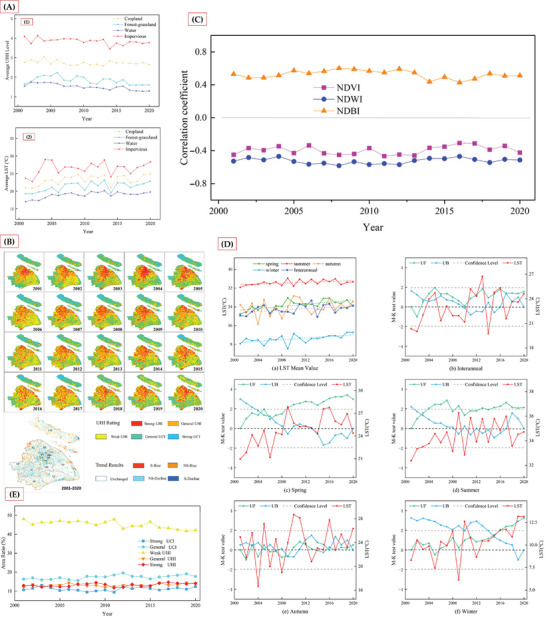
A) Trends of average Land Surface Temperature (LST) and Urban Heat Island (UHI) levels for different land use types (Cropland, Water, Forest‐grassland, Impervious surfaces) from 2000 to 2020.^[^ ^32^ ^]^ B) UHI classification maps for different years (2001‐2020), showing the spatial distribution of Strong UHI (Urban Heat Island), General UHI, and Strong UCI (Urban Cold Island).^[^ ^32^ ^]^ C) Correlation coefficients between LST and NDVI (Normalized Difference Vegetation Index), NDWI (Normalized Difference Water Index), and NDBI (Normalized Difference Built‐up Index) from 2000 to 2020.^[^ ^32^ ^]^ D) Seasonal variations of LST and UHI trends for spring, summer, autumn, and winter.^[^ ^32^ ^]^ E) Annual percentage of the area covered by different UHI levels (e.g., Strong UHI, General UHI, Weak UHI, Strong UCI) from 2005 to 2020.^[^ ^32^ ^]^

The primary cooling mechanism is evapotranspiration and evaporative cooling. Plants release water vapor through leaf stomata into the atmosphere, a process that absorbs substantial heat energy.^[^ ^33^ ^]^ As shown in **Figure** **7**A, forest‐grassland areas maintain an average surface temperature of 21.19 °C, significantly lower than the 26.47 °C observed in impervious surfaces, demonstrating vegetation's pronounced cooling effect. Evaporative cooling occurs not only through plant leaves but also across soil surfaces and vegetated areas, creating a multi‐layered cooling process.^[^ ^34^ ^]^

Shading represents the second crucial mechanism. Plant canopies intercept and disperse solar radiation, reducing direct surface heating.^[^ ^35^ ^]^ Figure 7B reveals Shanghai's urban heat island spatial distribution, showing notably weaker heat island intensity in vegetated areas (western waterways and northern regions). Trees and vegetation not only directly block sunlight but also alter the surface radiation balance, reducing heat absorption and accumulation.^[^ ^36^ ^]^

High albedo constitutes another critical mechanism. Unlike low‐reflectivity artificial surfaces such as asphalt and concrete, vegetation reflects more solar radiation.^[^ ^37^ ^]^ Figure 7C demonstrates the negative correlation (coefficient ‐0.4236) between the Normalized Difference Vegetation Index (NDVI) and surface temperature, confirming vegetation's significant role in temperature reduction. Green vegetation effectively reduces surface solar radiation absorption through its unique spectral properties.

Vegetation also moderates local microclimates by reducing wind speed, decreasing heat transfer, and increasing air humidity.^[^ ^38^ ^]^ Higher moisture content in air increases its heat capacity, helping stabilize local temperatures. Figure 7D shows relatively stable temperature trends in vegetated areas from 2001 to 2020, reflecting vegetation's temperature buffering capacity.

The vertical structure of vegetation significantly influences its cooling effectiveness. Multi‐layered vegetation structures combining trees, shrubs, and herbaceous plants provide comprehensive cooling effects. Different vegetation types contribute uniquely to cooling: trees, with their large canopies and strong transpiration capacity, typically offer the most significant cooling, while shrubs and herbaceous plants play vital roles in confined urban spaces.^[^ ^39^, ^40^ ^]^

Research^[^ ^32^ ^]^ also indicates a significant negative correlation between urban heat island intensity and urban‐rural vegetation cover differences. Figure 7E illustrates trends across different heat island intensity zones, showing how vegetation coverage effectively reduces urban‐rural temperature disparities, thereby weakening the heat island effect.

Despite vegetation restoration's crucial role in mitigating heat island effects, implementation faces numerous challenges. Limited urban space, water resource constraints, and soil conditions all affect restoration outcomes. Therefore, future urban greening strategies must comprehensively consider vegetation type selection, spatial layout, and maintenance management to develop scientific, systematic green infrastructure plans.

**Figure 8:**
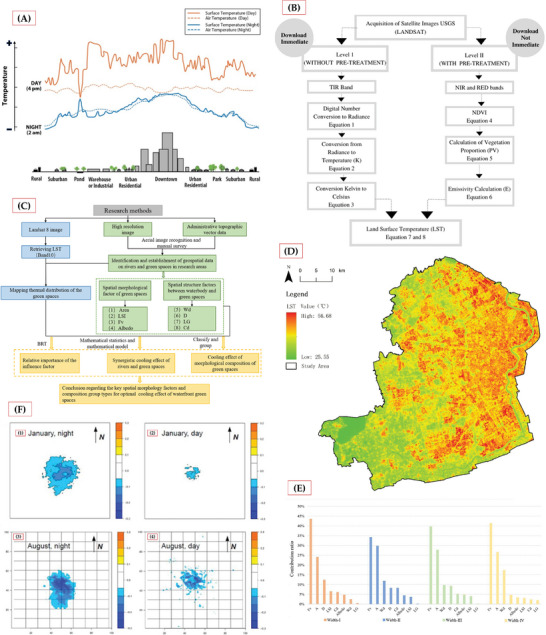
A) Temperature profiles showing Urban Heat Island (UHI) formation across regions.^[^ ^41^ ^]^ B) Land Surface Temperature (LST) data processing workflow.^[^ ^41^ ^]^ C) Research framework showing the integration of remote sensing, high‐resolution imagery, and topographic data to analyze spatial morphology‐temperature relationships.^[^ ^42^ ^]^ D) LST distribution map (25.55–56.68 °C), with red indicating higher temperatures and green indicating lower temperatures.^[^ ^42^ ^]^ E) Contribution rates of spatial variables (vegetation coverage *Fv*, area) to LST across river width levels.^[^ ^42^ ^]^ F) Solar panels' cooling effects on Paris's urban heat island in January and August (day/night), with darker blue showing stronger cooling. *N* indicates North. Color bars show temperature variations (*K*).^[^ ^43^ ^]^

Future research priorities include:

1. Future research should focus on several key directions to deepen our understanding of vegetation's role in heat island mitigation. Multi‐scale analysis^[^ ^41^ ^]^ has provided new perspectives by combining various data sources – primarily satellite remote sensing (Landsat 68.39%, MODIS 31.95%, ASTER 6.22%) along with ground stations (7.08%), field observations (2.42%), and UAV thermal sensing (2.25%). **Figure** **8**A demonstrates significant diurnal temperature variations from rural to urban centers, while Figure 8B outlines the complex LST data processing requirements including atmospheric correction and radiometric calibration. Research reveals that cooling effects vary by scale – large parks don't necessarily outperform smaller green spaces, with effectiveness depending more on spatial configuration and structural characteristics.
2. Long‐term monitoring proves crucial for assessing vegetation restoration's sustained impact and adaptability. The research framework shown in Figure 8C integrates Landsat 8 imagery, high‐resolution data, and administrative topographic vectors to analyze relationships between green space morphology and surface temperature.^[^ ^42^ ^]^ Figure 8D′s LST distribution map (25.55 °C‐56.68 °C) reveals significant spatial temperature variations. BRT model analysis (Figure 8E) identifies vegetation coverage (*Fv*) and area as primary cooling factors, contributing 32.7‐49.2% and 24.0‐29.5% respectively, with higher connectivity (*Cd*) correlating to enhanced cooling effects.
3. Comprehensive measure assessment represents another crucial research direction, as single vegetation restoration measures may have limitations. Masson et al.’s study^[^ ^43^ ^]^ demonstrates this through simulations of solar panel deployment combined with building characteristics and urban morphology. Figure 8F shows temperature reductions of 0.2*K* during daytime and 0.3*K* at night in dense urban areas. This variation suggests the need for multi‐variable analysis methods to evaluate measure synergies, considering implementation costs, community acceptance, and policy support.

In sum, vegetation restoration's impact extends beyond simple temperature reduction to encompass multiple ecosystem services. Future research must combine multi‐scale observation, long‐term monitoring, and comprehensive assessment to explore vegetation restoration's role in complex urban ecosystems. This approach will deepen our understanding of heat island effects and provide scientific guidance for global urban ecological restoration, laying the foundation for sustainable urban development.

### 4.2 Research Limitations and Significance

This multi‐case investigation has illuminated vegetation restoration's vital role in mitigating urban heat island effects across diverse contexts, while also revealing several areas warranting deeper exploration. Our methodology primarily relied on MOD13Q1 NDVI data at 250 m resolution, which, while providing valuable insights, limited our ability to capture fine‐grained urban microclimate variations and subtle vegetation changes. Though the two‐decade span from 2000 to 2020 offered robust temporal coverage, the annual sampling frequency may have missed important non‐linear dynamics in rapidly urbanizing areas. Future research would benefit from incorporating higher‐resolution data sources like Sentinel‐2 and PlanetScope, alongside dense ground sensor networks.

The study's focus on vegetation cover and heat island relationships, while productive, necessarily left some environmental variables less thoroughly explored. Factors such as soil moisture, wind patterns, precipitation, and air quality can significantly modulate vegetation's cooling effectiveness. For instance, moisture‐stressed soil can diminish evapotranspiration benefits, while local wind conditions may either enhance or suppress cooling effects. A more comprehensive analysis framework incorporating surface energy balance, microclimate dynamics, and socioeconomic variables would provide richer insights into these complex interactions.

Our case selection, encompassing Dexing City, Qibaoshan Township, and Dartford Ebbsfleet Garden City, offered valuable comparative perspectives but also highlighted the challenges of generalization across diverse urban contexts. Each site presents unique combinations of climate patterns, policy frameworks, and ecological characteristics. Tropical urban areas face fundamentally different challenges from temperate regions, while arid zones must navigate severe water constraints. Expanding the geographic scope to include more climate zones and socioeconomic contexts would strengthen our understanding of both universal principles and region‐specific optimization strategies.

Nevertheless, this research makes significant contributions by establishing a systematic framework that integrates multiple mechanisms of vegetation restoration and heat island mitigation. By revealing the synergistic relationships between evapotranspiration, albedo modification, shading effects, and microclimate regulation, we provide novel insights for both theoretical advancement and practical implementation. The study demonstrates how vegetation restoration delivers benefits beyond temperature reduction, encompassing air quality improvement, biodiversity enhancement, and urban landscape value elevation.

Looking forward, this integrative framework opens new avenues for investigating other ecosystem functions, from carbon sequestration to precipitation regulation and public health benefits. This expanded perspective supports the development of comprehensive urban sustainability strategies that balance economic growth with ecological preservation. By continuing to build on these findings, we can provide increasingly sophisticated scientific guidance for urban ecosystem management worldwide, ultimately contributing to more resilient and livable cities in the face of global environmental change.

## 5 Conclusion

This study explores the relationship between vegetation restoration and urban heat island mitigation across three mining regions, revealing both successes and implementation challenges. Analysis of Qibaoshan Township, Dexing City, and Dartford from 2000–2020 demonstrates that systematic vegetation restoration effectively reduces local temperatures and improves urban livability.

Our research reveals that vegetation moderates urban heat through multiple mechanisms. Plant canopies provide crucial shading and evaporative cooling, with forested areas maintaining temperatures 2–3 °C lower than exposed surfaces. The vertical structure of vegetation significantly impacts cooling effectiveness, with multi‐layered combinations of trees, shrubs, and ground cover providing optimal temperature regulation.

The case studies highlight different approaches to ecological restoration. Qibaoshan Township achieved substantial heat island reduction through comprehensive mine tailings reclamation and vegetation regrowth. Dexing City maintained environmental stability by transitioning from open‐pit to underground mining while implementing systematic greening initiatives. Dartford's transformation from quarry to garden city demonstrates both the potential and challenges of integrating ecological restoration with urban development.

While our findings support vegetation restoration as an effective strategy for heat island mitigation, methodological limitations suggest areas for future research. Higher resolution remote sensing data, expanded geographical sampling, and integration of socioeconomic factors would strengthen understanding of these complex urban‐ecological interactions. Nevertheless, this study provides valuable guidance for mining regions worldwide, demonstrating that scientific planning, policy support, and innovative technology can successfully balance economic development with environmental protection.

## Conflict of Interest

The authors declare no conflict of interest.

## Author Contributions

H.Y. and I.Z. conceptualized the study. H.Y. was responsible for the methodology, formal analysis, resources, data curation, original draft preparation, and visualization. I.Z. validated the work and supervised the project, while also contributing to writing—review and editing. C.M.F. also participated in the writing—review and editing and provided supervision. Finally, I.Z. managed the project administration. All authors have read and agreed to the published version of the manuscript.

## Data Availability

The Fractional Vegetation Cover (FVC) statistics reported in this paper are sourced from the MOD13Q1 data product (with a spatial resolution of 250 m), which is available through the NASA Earth Observing System Data and Information System (EOSDIS). The temperature statistics presented in this paper are based on the MOD11A2 product (with a 1 km spatial resolution), which is a Moderate Resolution Imaging Spectroradiometer (MODIS) Terra Land Surface Temperature and Emissivity 8‐day data set. All data have been uploaded: https://doi.org/10.13140/RG.2.2.21021.63209.

## References

[1] S. Chatterjee , K. Gupta , India. Remote Sensing Applications: Society and Environment 2021, 23, 100581. 10.1016/j.rsase.2021.100604 PMC9764503 36568403

[2] R. Sarda , S. Pal , In Advancements in Urban Environmental Studies: Application of Geospatial Technology and Artificial Intelligence in Urban Studies, Springer International Publishing, Cham, 2023, pp. 301–324.

[3] Y. Xu , W. Hou , C. Zhang , Land 2023, 12, 1701.

[4] S. Labant , M. Bindzarova Gergelova , Z. Kuzevicova , S. Kuzevic , G. Fedorko , V. Molnar , Minerals 2020, 10, 489.

[5] B. Halder , J. Bandyopadhyay , K. M. Khedher , C. M. Fai , F. Tangang , Z. M. Yaseen , Environ. Sci. Pollut. Res. 2022, 29, 73147. 10.1007/s11356-022-20821-x 35624371

[6] Z. A. Rahaman , A. A. Kafy , M. Saha , A. A. Rahim , A. I. Almulhim , S. N. Rahaman , Md. A. Fattah , M. T. Rahman , Kalaivani S , Abdullah‐Al‐Faisal , A. Al Rakib , Malaysia. Building and Environment 2022, 222, 109335.

[7] K. Deilami , M. Kamruzzaman , J. F. Hayes , Remote Sensing 2016, 8, 716.

[8] A. Esposito , G. Pappaccogli , A. Donateo , P. Salizzoni , G. Maffeis , T. Semeraro , J. L. Santiago , R. Buccolieri , Remote Sens 2024, 16, 4496.

[9] C. B. Jayasinghe , N. C. Withanage , P. K. Mishra , K. Abdelrahman , M. S. Fnais , Sustainability 2024, 16, 10635.

[10] H. Yu , I. Zahidi , Sci. Total Environ. 2023, 859, 160392. 36423851 10.1016/j.scitotenv.2022.160392

[11] H. Yu , G. Gunaratna , I. Zahidi , C. M. Fai , The Innovation 2024, 5, 100578. 38445020 10.1016/j.xinn.2024.100578 PMC10912676

[12] H. Yu , I. Zahidi , D. Liang , China. Environmental research 2023, 225, 115634. 36889570 10.1016/j.envres.2023.115634

[13] H. Yu , I. Zahidi , D. Liang , Environ. Res. 2023, 225, 115613. 36870554 10.1016/j.envres.2023.115613

[14] https://www.ndrc.gov.cn/fggz/dqzx/zyxdqzxfz/202210/t20221024_1338985.html (accessed: September 28, 2024).

[15] https://www.icswb.com/h/100075/20150915/346706.html (accessed: September 28, 2024).

[16] H. Yu , I. Zahidi , C. M. Fai , Environ. Res. 2023, 232, 116336. 37321336 10.1016/j.envres.2023.116336

[17] https://ebbsfleetgardencity.org.uk/ebbsfleet‐garden‐city/the‐vision (accessed: September 28, 2024).

[18] https://healthyurbanism.net/ebbsfleet‐garden‐city (accessed: September 28, 2024).

[19] https://ebbsfleetgardencity.org.uk/support‐our‐vision/environment (accessed: September 28, 2024).

[20] L. Liu , B. Yao , Transactions of the Chinese Society of Agricultural Engineering 2010, 26, 230.

[21] K. Yan , S. Gao , H. Chi , J. Qi , W. Song , Y. Tong , X. Mu , G. Yan , IEEE Transactions on Geoscience and Remote Sensing 2021, 60, 1.

[22] S. Huang , L. Tang , J. P. Hupy , Y. Wang , G. Shao , Journal of Forestry Research 2021, 32, 1.

[23] L. Yang , K. Jia , S. Liang , M. Liu , X. Wei , Y. Yao , X. Zhang , D. Liu , Remote Sens. 2018, 10, 549.

[24] D. X. Song , Z. Wang , T. He , H. Wang , S. Liang , Science of Remote Sensing 2022, 6, 100058.

[25] M. D. de Andrade , R. C. Delgado , S. J. M. da Costa de Menezes , R. de Ávila Rodrigues , P. E. Teodoro , C. A. da Silva Junior , M. G. Pereira , Environmental Monitoring and Assessment 2020, 193, 1. 33410968 10.1007/s10661-020-08788-z

[26] A. Rasul , H. Balzter , C. Smith , J. Remedios , B. Adamu , J. A. Sobrino , M. Srivanit , Q. Weng , Land 2017, 6, 38.

[27] B. Alahmad , L. P. Tomasso , A. Al‐Hemoud , P. James , P. Koutrakis , International Journal of Environmental Research and Public Health 2020, 17, 2993. 32357399 10.3390/ijerph17092993 PMC7246769

[28] H. Yu , I. Zahidi , M. F. Chow , Iscience 2020, 26, 107667. 10.1016/j.isci.2023.107667 PMC10481345 37680487

[29] S. Xie , C. Yu , B. Peng , H. Xiao , W. Zhang , Z. Zhou , M. E. Åström , International Journal of Environmental Science and Technology 2022, 19, 10707.

[30] X. Liu , H. R. Fan , N. J. Evans , G. E. Batt , B. I. McInnes , K. F. Yang , K. Z. Qin , SE China: Insights From Geo‐and Thermochronology Mineralium Deposita 2014, 49, 809.

[31] I. Pluchinotta , A. Pagano , T. Vilcan , S. Ahilan , L. Kapetas , S. Maskrey , V. Krivtsov , C. Thorne , E. Donnell , Sustainable Cities and Society, 2021, 67, 102709.

[32] M. Wang , H. Lu , B. Chen , W. Sun , G. Yang , Remote Sens. 2023, 15, 3732.

[33] S. Cascone , J. Coma , A. Gagliano , G. Pérez , Build. Environ. 2019, 147, 337.

[34] M. Shen , P. Shilong , J. Su‐Jong , L. Zhou , Z. Zhenzhong , C. Philippe , C. Deliang , et al., Proc. Natl. Acad. Sci. USA 2015, 112, 9299.26170316

[35] M. A. Rahman , V. Dervishi , A. Moser‐Reischl , F. Ludwig , H. Pretzsch , T. Rötzer , S. Pauleit , Comparative analysis of shade and underlying surfaces on cooling effect, Vol. 63, Urban Forestry & Urban Greening, Elsevier, Netherlands, 2021, p. 127223.

[36] H. Huryna , J. Pokorný , Folia geobotanica 2016, 51, 191.

[37] C. A. Gueymard , V. Lara‐Fanego , M. Sengupta , Y. Xie , Sol. Energy 2019, 182, 194.

[38] S. Fan , M. Zhang , Y. Li , K. Li , L. Dong , China. Sustainability 2021, 13, 4791.

[39] G. Rutten , A. Ensslin , A. Hemp , M. Fischer , PLoS One 2021, 10, e0138822. 10.1371/journal.pone.0138822 PMC4583428 26406985

[40] J. Yan , J. Zhang , Q. Wang , X. He , H. Zheng , Stand Structural Characteristics Determine Ecosystems Multifunctionality of Urban Forests in Changchun City, Northeast China, Urban Forestry & Urban Greening, Elsevier, Netherlands, 2024, p. 128647.

[41] C. R.d. Almeida , A. C. Teodoro , A. Gonçalves , Environments 2021, 8, 105.

[42] Y. Jiang , J. Huang , T. Shi , H. Wang , Int. J. Environ. Res. Public Health 2021, 18, 11404. 34769917 10.3390/ijerph182111404 PMC8583193

[43] V. Masson , M. Bonhomme , J.‐L. Salagnac , X. Briottet , A. Lemonsu , Frontiers in Environmental Science 2014, 2, 14.

